# Haemodynamic responses to head‐up tilt versus lower‐body negative pressure in type 1 diabetes

**DOI:** 10.1113/EP093588

**Published:** 2026-07-28

**Authors:** Victorien Faivre‐Rampant, Jules Warnier, Perrine Larmet, Damien Garcia, Michael Joubert, Damian Miles Bailey, Igor Mekjavic, Hervé Normand, Amir Hodzic

**Affiliations:** ^1^ Inserm Comete, GIP Cyceron Normandie Univ, CHU Caen Normandie Caen France; ^2^ Department of Automatics, Biocybernetics, and Robotics Jozef Stefan Institute Ljubljana Slovenia; ^3^ Jozef Stefan International Postgraduate School Ljubljana Slovenia; ^4^ Department of Cardiology, Inserm Comete, GIP Cyceron Normandie Univ, CHU Caen Normandie Caen France; ^5^ INSERM research director at Creatis (Medical Imaging Research Laboratory) University of Lyon Lyon France; ^6^ Diabetes Care Unit Caen University Hospital, UNICAEN Caen France; ^7^ Neurovascular Research Laboratory Faculty of Life Sciences and Education University of South Wales Pontypridd UK

**Keywords:** fluid shift, haemodynamics, head‐up tilt, lower‐body negative pressure, orthostatic stress, type 1 diabetes

## Abstract

Head‐up tilt (HUT) and lower‐body negative pressure (LBNP) both reduce central blood volume but differ in heart orientation and regional fluid shifts. Whether they evoke comparable cardiovascular regulation when matched for preload remains uncertain. We characterized differences in fluid redistribution and haemodynamic responses during graded HUT and LBNP at intensities eliciting equivalent reductions in thoracic blood volume (TBV). In a randomized crossover design, 30 young adults (16 healthy control subjects and 14 with type 1 diabetes) underwent both HUT (22°, 42°, 58° and 80°) and LBNP (−10, −20, −35 and −50 mmHg). Segmental bioimpedance, haemodynamic and ECG responses were recorded continuously. Equivalent reductions in TBV were obtained for 22° vs. −10 mmHg, 42° vs. −20 mmHg, and 80° vs. −35 mmHg (all *P* > 0.9). However, regional fluid shifts differed, in that HUT induced splanchnic pooling, whereas LBNP promoted pelvic pooling. At matched reductions in TBV, stroke volume decreased more during HUT than during LBNP (*P* = 0.016). Cardiac output was maintained during HUT owing to more pronounced tachycardia, but it declined during LBNP at −35 mmHg. Type 1 diabetics exhibited consistently higher heart rate across all conditions, but preserved stroke volume, cardiac output and mean arterial pressure relative to control subjects. Although HUT and LBNP generate comparable reductions in TBV, they elicit distinct cardiovascular adjustments. HUT engages gravitational influences that help to maintain cardiac output, whereas LBNP isolates baroreflex‐mediated responses, underscoring the unique contribution of gravity to orthostatic regulation. Collectively, these findings refine interpretation of orthostatic models and highlight their utility for detecting early reductions in autonomic reserve in type 1 diabetes.

## Abstract (highlights)

- **What is the central question of this study?**
  To what extent do head‐up tilt (HUT) and lower‐body negative pressure (LBNP), when matched for equivalent reductions in central blood volume, elicit comparable patterns of fluid redistribution and cardiovascular regulation, with a specific focus on type 1 diabetes?
- **What is the main finding and its importance?**
  Despite similar preload reductions, the modalities yielded distinct responses: HUT promoted splanchnic pooling and maintained cardiac output, whereas LBNP induced pelvic pooling and reduced cardiac output. These differences underscore the influence of gravity on cardiovascular control and highlight the value of orthostatic testing for detecting early autonomic impairment.

## 1 INTRODUCTION

Cardiovascular regulation depends crucially on gravitational loading and venous return (Caiani et al., 2009; Negishi et al., 2017). During the transition from supine to upright posture, gravity induces blood pooling in the lower extremities, thereby reducing venous return, cardiac preload and stroke volume (SV). Head‐up tilt (HUT) and lower‐body negative pressure (LBNP) are well‐established experimental models that reproduce orthostatic stress, yet they impose haemodynamic strain through distinct mechanisms: HUT engages gravity and modifies cardiac orientation, whereas LBNP decreases thoracic blood volume (TBV) via suction below the diaphragm while maintaining a horizontal posture. Although often assumed to be equivalent physiologically, important differences between the two remain unresolved.

Prior work has shown that HUT predominantly produces splanchnic pooling, whereas LBNP promotes pelvic pooling, implying that body orientation and pressure distribution shape venous capacitance and vascular control beyond the reduction in TBV alone (Taneja et al., 2007). Additionally, experimental cardiac models demonstrate that gravity can impose an intraventricular hydrostatic pressure gradient of ∼6 mmHg from base to apex, potentially influencing ventricular filling and global cardiac performance (Pantalos et al., 1998, 2005). However, its relevance to human cardiovascular regulation across gravitational orientations remains poorly defined.

Beyond these haemodynamic considerations, type 1 diabetes (T1D) represents a particularly relevant model to investigate early cardiovascular dysregulation, not only because of emerging myocardial alterations (Hodzic et al., 2016; Joubert et al., 2019; Kiencke et al., 2010; Markuszewski et al., 2006) but also owing to the high prevalence of subclinical autonomic nervous system impairment (Williams et al., 2022). Cardiac autonomic neuropathy, a frequent and underdiagnosed complication of diabetes, is characterized by a progressive imbalance between parasympathetic and sympathetic activity, which can occur early in the disease course, even in the absence of overt cardiovascular disease (Williams et al., 2022).

The natural history of cardiac autonomic neuropathy is marked by an initial length‐dependent neuropathy of the vagus nerve, leading to early parasympathetic withdrawal and reduced heart rate variability, followed by relative sympathetic predominance and resting tachycardia. As the condition progresses, sympathetic fibres are also affected, resulting in impaired baroreflex responses and orthostatic hypotension, and ultimately in a denervated heart with fixed heart rate (Williams et al., 2022). Importantly, this autonomic imbalance develops well before clinically apparent cardiac dysfunction and is associated with increased cardiovascular risk.

In light of these data, the present study was designed to examine patterns of fluid redistribution and haemodynamic responses during graded HUT and LBNP, initially identifying intensities that produced equivalent reductions in preload, then characterizing dose–response relationships and group differences between healthy and T1D participants.

## 2 MATERIALS AND METHODS

### 2.1 Ethics

The study was approved by the French National Ethics Committee (Comité de Protection des Personnes Nord‐Ouest VI; reference 2023‐A00869‐36) and conducted in accordance with the *Declaration of Helsinki* and European regulations. The protocol was registered at ClinicalTrials.gov (NCT06190756). All participants provided written informed consent after receiving a full explanation of the study and were financially compensated for their time and travel.

### 2.2 Participants

A total of 32 volunteers (16 males and 16 females) were recruited and assigned to two groups: a control group (Ctrl) of healthy individuals (*n* = 16, 8 males and 8 females) and a group with T1D (*n* = 14, 7 males and 7 females). Participants in the T1D group were prescreened among patients followed at the University Hospital Centre of Caen Normandy. All participants were between 18 and 40 years of age. This age range was chosen to minimize the risk of silent myocardial ischaemia in both populations. A detailed list of inclusion and exclusion criteria is provided in the Supplementary material (Table S1). Female participants were tested outside of their menstrual period (3 without hormonal contraception in the follicular phase and 12 with hormonal contraception in the hormonal phase).

### 2.3 Design

To establish dose–response curves and assess how gravitational orientation affects cardiac performance, haemodynamic responses and fluid distribution data were collected at four levels of LBNP relative to the ambient pressure and four HUT angles. Changes in thoracic impedance were used as an index of preload reduction, allowing identification of LBNP and HUT intensities that produced comparable decreases in TBV. Cardiovascular responses were then compared between these matched conditions to isolate the additional effects of upright posture and the gravitational vector along the cardiac axis, independent of preload.

### 2.4 Measurements

**FIGURE 1:**
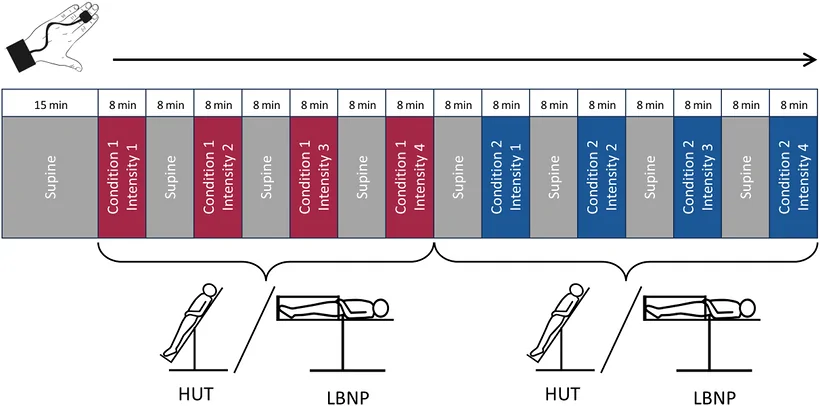
Schematic representation of the experimental protocol. Participants underwent head‐up tilt (HUT) and lower‐body negative pressure (LBNP) in a randomized order, refered as condition 1 and condition 2 (red and blue blocks indicate the two conditions, while the grey blocks represent supine periods). Intensities were 22°, 42°, 58° and 80° for HUT, and −10, −20, −35 and −50 mmHg for LBNP.

Non‐invasive continuous haemodynamic monitoring was performed via digital photoplethysmography (Finapres NOVA, Finapres Medical Systems BV, The Netherlands). The degree of central hypovolaemia induced by both HUT and LBNP has a rapid onset and reversal, with ∼50% of venous blood pooling occurring within 25–40 s (Crystal & Salem, 2015; Lindenberger & Länne, 2015). Standard protocols typically use short stages (3–6 min), with haemodynamic data averaged after 1 or 2 min (Fu et al., 2009). Therefore, we chose to set all conditions (HUT or LBNP) to 8 min exposures, followed by an 8 min recovery period in the horizontal supine position to allow haemodynamic variables to return to baseline. A 2 min stabilization phase preceded each exposure and recovery period, during which no data were collected. Haemodynamic and impedance signals were recorded continuously throughout both the exposure and recovery phases. The overall experimental sequence is illustrated in Figure 1.

Additional echocardiographic data were acquired as part of the experimental protocol but are not presented in the present manuscript and will be reported separately.

### 2.5 HUT

**FIGURE 2:**
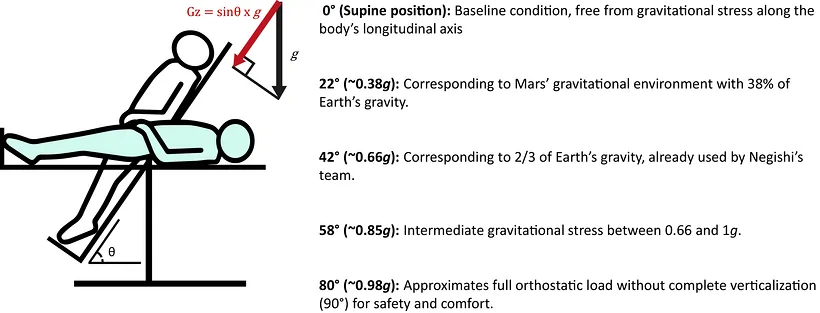
The partial gravitational load or the hydrostatic gradient from head to foot (*Gz*) is equal to the sine of the head‐up tilt angle (sinθ) multiplied by the gravitational acceleration (*g*). Five angles were selected for the protocol: 0°, 22°, 42°, 58° and 80° (Negishi et al., 2017).

As a reference for orthostatic stress testing, HUT represents the primary experimental modality to explore the effect of gravitational loading on the cardiovascular system. In this study, a custom‐designed tilt + LBNP table was tilted manually to the desired angles. Precise angular positioning was achieved using steel cables of calibrated length and verified with an inclinometer (DOG2 MEMS, Measurement Specialties Inc., Hampton, VA, USA) with an accuracy of 0.2°. Participants were supported on a bicycle saddle, avoiding reliance on leg muscles, with the LBNP chamber directly mounted to the tilt table to maintain identical positioning across all conditions. Using a saddle in the HUT condition minimized weight‐bearing and muscle contractions that could interfere with impedance measurements, which are highly sensitive to muscle activity. Consequently, both LBNP and HUT protocols were conducted in non‐weight‐bearing conditions, consistent with prior observations (Ogoh et al., 2003). Additionally, saddle support during orthostatic testing has been shown to reduce variability in cardiovascular responses and the risk of orthostatic hypotension (Fitzpatrick et al., 1991; Murray et al., 1966). The selected HUT angles were derived using trigonometric calculations to provide a progressive increase in gravitational stress (*g*) from supine (0°) to near‐upright (80°) posture (Negishi et al., 2017) (Figure 2).

### 2.6 LBNP

LBNP reproduces the orthostatic‐like cardiovascular stress without altering heart orientation or introducing a vertical gravitational vector along the cardiac axis. LBNP was generated with a vacuum pump, and the pressure within the LBNP chamber was monitored with a manometer (EE600, E+E Electronik GES, Engerwitzdorf, Germany). Pressure inside the LBNP chamber was adjusted manually using a three‐way valve. The seal between the LBNP chamber and the participants was achieved with a neoprene skirt. Following the literature, the skirt was adjusted to provide a seal at the iliac crest level. This positioning allows effective pooling of blood within the lower limbs and pelvic venous compartments, thereby inducing central hypovolaemia while minimizing potential confounding effects on intra‐abdominal pressure and respiratory mechanics (Crystal & Salem, 2015; Goswami et al., 2019).

To establish dose–response curves, four LBNP intensities (−10, −20, −35 and −50 mmHg) were selected based on previous studies to induce graded orthostatic stresses up to −50 mmHg, which is generally considered to approximate the cardiovascular load observed at 1*g* (Caiani et al., 2007, 2009). The experiment was terminated in the event of presyncopal symptoms or any other termination criteria determined by the European Space Agency standard operating procedure for measurement of orthostatic tolerance (EI‐Bedawi & Hainsworth, 1994; Guinet et al., 2009).

### 2.7 Order of experimental conditions

**FIGURE 3:**
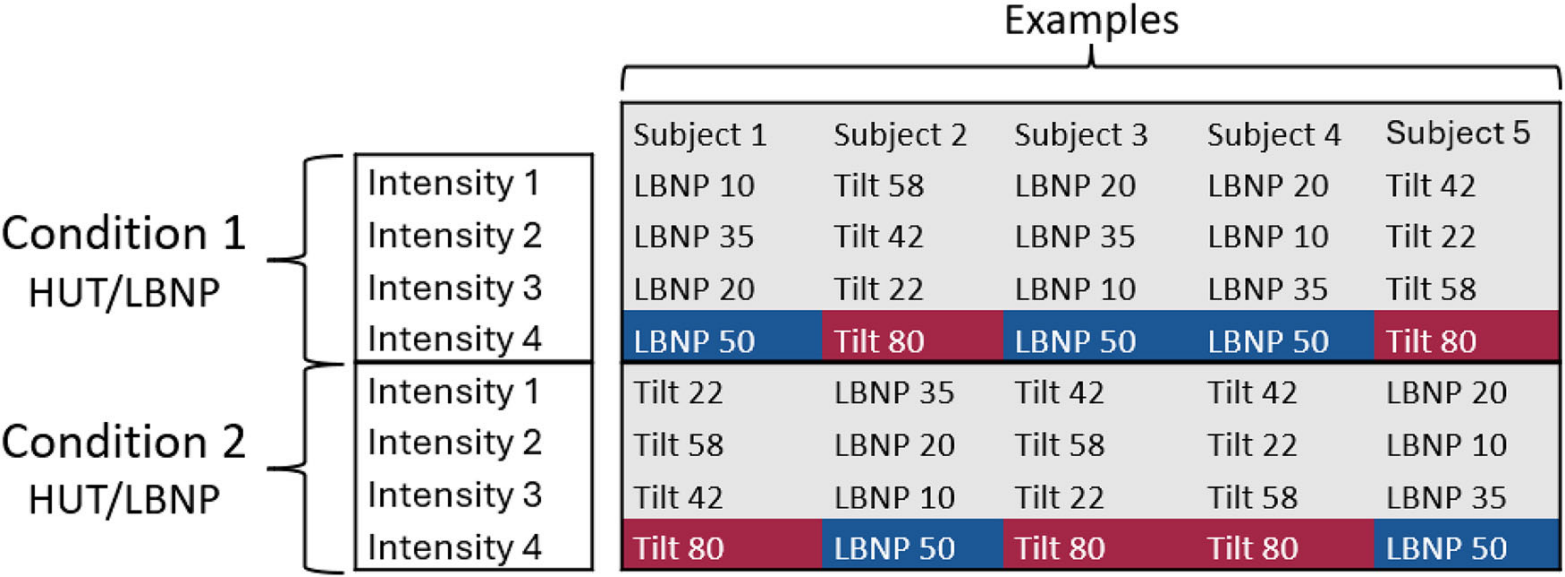
Examples of randomized sequences for the order of experimental conditions. Abbreviations: HUT, head‐up tilt; LBNP, lower‐body negative pressure.

To minimize methodological bias, the order of experimental conditions was randomized in a single‐blind manner. For each participant, the initial intervention (HUT or LBNP) was assigned randomly, as was the subsequent sequence of angles and pressures within each trial. To preserve physiological comparability, the same sequence of intensity progression was applied for both HUT and LBNP. Each condition was separated by a full supine recovery phase designed to restore baseline parameters. Using identical sequences across modalities prevented the risk of ordering effects. In addition, because individuals with T1D can have reduced orthostatic tolerance, the most demanding stages (80° HUT and −50 mmHg LBNP) were performed last to avoid presyncope and ensure completion of all preceding conditions (Figure 3).

### 2.8 Data acquisition

#### 2.8.1 Impedance

**FIGURE 4:**
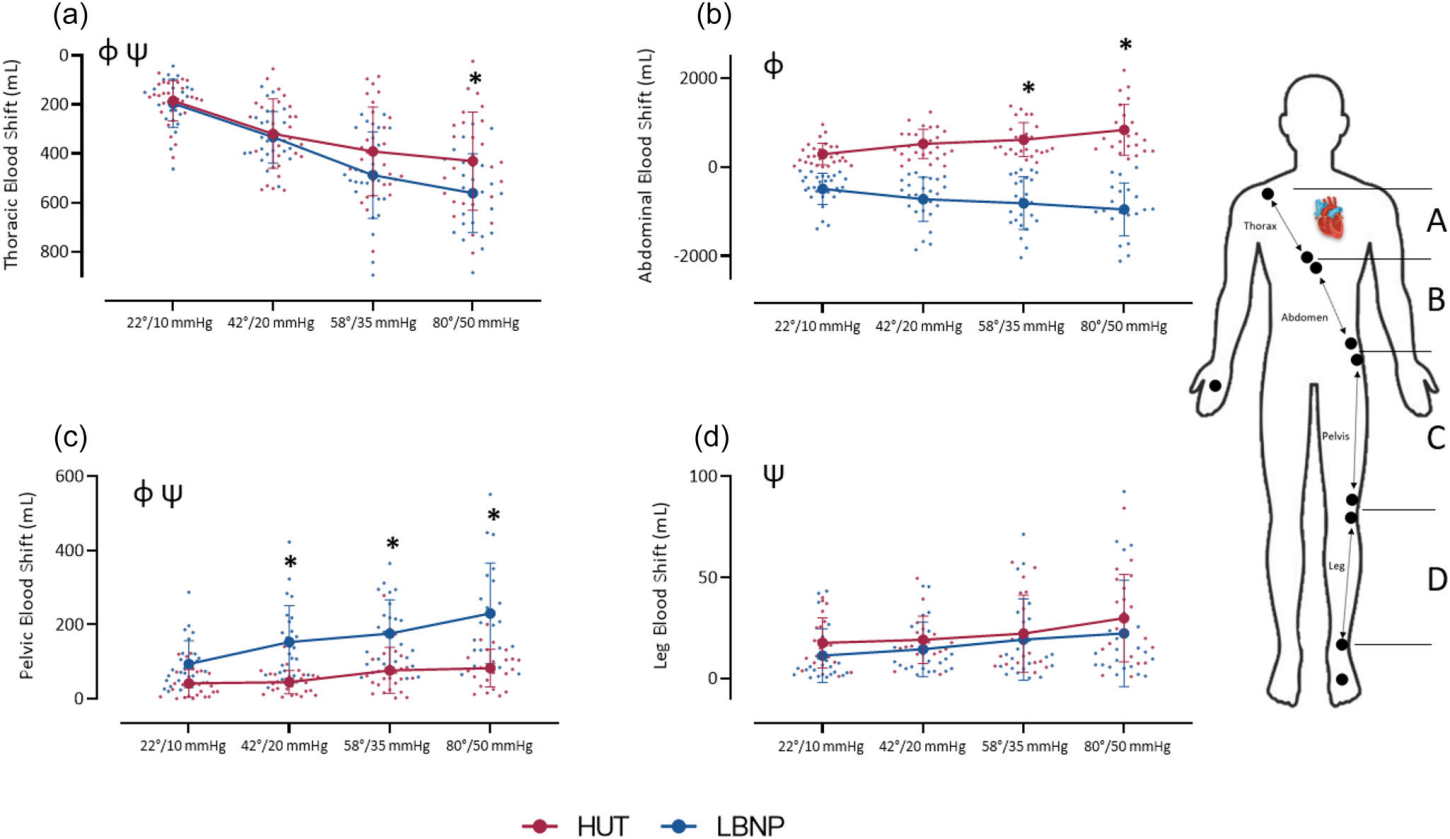
Schematic representation of the blood shift (in millilitres) relative to the supine position measured through segmental bioimpedance during four angles of head‐up tilt (HUT) and four intensities of lower‐body negative pressure (LBNP): (A) thoracic fluid shift; (B) abdominal fluid shift; (C) pelvic fluid shift; and (D) leg fluid shift. Error bars indicate the SD. ^φ^Significant differences between HUT and LBNP. ^ψ^Significant effect of the orthostatic stress intensity. ^*^Significant difference between modalities at a specific intensity. On the right is presented a schematic representation of the high‐resolution impedance meter using 10 electrodes to measure the fluid shift in the four areas.

Segmental bioimpedance was used to assess body fluid redistribution during the experimental conditions (Krantz et al., 2000; Matzen et al., 1991). A weak, high‐frequency electrical current was applied between hand and foot electrodes. The variation in impedance indicated shifts in blood volume within defined anatomical segments (Showkat et al., 2023; Stewart & Montgomery, 2004). Measurements of impedance were achieved with a high‐resolution impedance meter (UFI model 2994/D, UFI, Morro Bay, CA, USA), with 10 surface electrodes positioned according to the configuration illustrated in Figure 4. Four compartments were analysed, namely the thorax, abdomen, pelvis and leg.

To ensure reliable signal quality, specific time points were selected for impedance data analysis. Given the susceptibility of impedance measurements to external perturbations, sampling was performed immediately before and after the onset and offset of each HUT or LBNP condition. Given that impedance is sensitive to ventilation, participants were instructed to perform a short breath‐hold (∼10 s) at end expiration with open glottis, immediately before the change in condition, and another ∼30 s after the transition, allowing sufficient time for haemodynamic stabilization. By comparing impedance values before and after each change in condition, we can estimate the net fluid shift into or out of a given body compartment, as defined by the segmental electrode configuration.

Using the formula developed by Geddes and Sadler (1973), which incorporates blood resistivity and haematocrit, the estimated volume of blood shifting into or out of each segment (defined between two electrodes) can be calculated with the following formula (Geddes & Sadler, 1973; Taneja et al., 2007):

$$\Delta \mathrm{segmental}\;\mathrm{blood}\;\mathrm{volume}\;\;\left(\mathrm{mL}\right)=\rho \times \left(\frac{L^{2}}{R_{0}R_{1}}\right)\times \Delta R$$

where ρ is the electrical conductivity of the blood, estimated as 53.2 × e^(haematocrit × 0.022)^, *L* is the distance between the two electrodes of the measured segment, *R*_0_ is the resistance between the two electrodes before modification of HUT angle or LBNP intensity, *R*_1_ is the resistance between electrodes after modification of HUT angle or LBNP intensity, and Δ*R* = (*R*_1_ − *R*_0_).

#### 2.8.2 Arterial pressure and haemodynamics

Cardiovascular and haemodynamic parameters were assessed using non‐invasive continuous finger plethysmography (Finapres NOVA, Finapres Medical Systems BV, The Netherlands). Based on the continuous measurement of the finger arterial pressure, brachial systolic and diastolic pressures were measured and corrected with the waveform filtering and level correction (B. E. Westerhof et al., 2002) and adjusted further using a height correction unit to account for hydrostatic pressure differences between the finger and the heart, which are particularly relevant during tilt procedures. Haemodynamic indices, including heart rate (HR), SV, cardiac output (CO) and total peripheral resistance (TPR), were calculated using the Modelflow method (Wesseling et al., 1993; N. Westerhof et al., 2009). Data analysis was performed with sampling periods of 90–120 s during haemodynamic steady states at the end of each condition.

#### 2.8.3 ECG

A resting 12‐lead ECG was performed on each participant after inclusion in the study and before the start of the experimental sessions to screen for any previously undetected cardiac abnormalities that could contraindicate participation. During the experimental protocol, continuous ECG monitoring was conducted using a five‐lead configuration with a sampling frequency of 1000 Hz. This allowed real‐time surveillance of cardiac electrical activity throughout both the HUT and LBNP procedures and assessment of the HR response.

#### 2.8.4 Haematocrit

Upon completion of each experimental session, two capillary blood samples (2 × 200 µL) were collected from the fingertip using microcapillary tubes. The samples were immediately centrifuged at 3000*g* for 5 min to allow separation of blood components by density. Following centrifugation, haematocrit was determined as the ratio of the packed red blood cell column to the total blood column height, expressed as a percentage. The final haematocrit value used for analysis was the average of the two measurements. This parameter is used in calculations of fluid shift derived from bioimpedance measurements, because blood resistivity is inversely related to haematocrit according to Geddes’ model (Geddes & Sadler, 1973).

#### 2.8.5 Diabetes and glycaemic monitoring

For T1D patients, data regarding diabetes management were collected, including the most recent glycated haemoglobin value within the previous 6 months and continuous glucose monitor (CGM)‐derived metrics (time in range, time below range, time above range and glucose management indicator). During the experimental sessions, glycaemic stability was ensured through continuous glucose monitoring using each participant's personal CGM device. Glucose values displayed on the CGM reader were reviewed and documented every 15 min.

### 2.9 Statistical analysis

#### 2.9.1 Prospective power calculations and sample size estimates

To estimate the required sample size, we relied on data from Negishi et al. (2017), who also examined the cardiovascular responses to gravitational stress. Using transthoracic echocardiography at comparable angles (0, 22°, 42° and 80°), the authors demonstrated a reduction in SV from 63 ± 15 mL at 0° to 54 ± 16 mL at 80° (*P* < 0.001). Based on these differences and the corresponding effect size (Cohen's *d*) of 0.6, the present study required a (minimum) sample size of 12 participants per group (total sample size of 24) to achieve a power (1 − β) of 0.80 at *P* < 0.05 for all two‐tailed tests. We chose to inflate our final sample size to 32 participants (*n* = 16 per group) given the potential for loss to follow‐up and/or technical complications.

#### 2.9.2 Inferential statistics

Data were analysed using JASP software (JASP v.0.18.3, University of Amsterdam, The Netherlands). Repeated Shapiro–Wilks *W* tests were used to confirm distribution normality. Data were analysed using a three‐way mixed ANOVA (group: healthy vs. TD1) or sex (male vs. female) × condition: HUT vs. LBNP × intensity (22° of HUT/10 mmHg vs. 42° of HUT/20 mmHg vs. 58° of HUT/35 mmHg vs. 80° of HUT/50 mmHg). Following the identification of a significant interaction, *post hoc* analyses were performed using Bonferonni corrected pairwise comparisons (Student's paired *t*‐test within groups; Student's unpaired *t*‐test between groups). Significance was established at *P* < 0.05 for all two‐tailed tests (with individual *P*‐values for all comparisons), and data are expressed as the mean ± SD.

## 3 RESULTS

### 3.1 Demographics

Of the 32 participants enrolled, 30 (16 controls and 14 with T1D) completed the full protocol without presyncopal symptoms. Two T1D patients exhibited early symptoms, leading to early termination of the HUT testing, and were excluded from the final analysis.

The anthropometric, clinical and biochemical data of the population are detailed in Table 1. Among female participants, two were using oral contraception and three were in their luteal phase (defined as >16 days after the beginning of their last menstrual period).

**TABLE 1:** Participant demographics.

Parameter	Total	Ctrl	T1D	*P*‐value
	(*n* = 30)	(*n* = 16)	(*n* = 14)	
Sex	15 (50%)	8 (50%)	7 (50%)	–
Age (years)	28.2 ± 5.2	28.5 ± 5.3	27.9 ± 5.2	0.768
Stature (cm)	170.9 ± 8.4	171.4 ± 9.7	170.0 ± 6.9	0.747
Mass (kg)	69.9 ± 12.4	69.8 ± 13.9	69.9 ± 10.9	0.981
Body mass index (kg/m^2^)	23.8 ± 3.1	23.6 ± 2.7	24.1 ± 5.6	0.634
Haematocrit (%)	44.5 ± 3.4	44.0 ± 3.6	45.2 ± 3.2	0.323
Resting HR (beats/min)	68.9 ± 11.9	66.2 ± 13.0	72.18 ± 9.9	0.185
Resting MAP (mmHg)	100.2 ± 13.4	101.9 ± 12.2	98.1 ± 14.9	0.257
T1D duration (years)	–	–	16,7 ± 5	–
HbA1c (%)	–	–	7.4 ± 1.7	–
eGFR (CKD‐EPI) (mL/min/1.73 m²)	–	–	114.6 ± 10.7	–
Albuminuria/creatininuria ratio (mg/mmol)	–	–	4.9 ± 9.3	–
Time in range (%)	–	–	63.8 ± 12.9	–
Time below range (%)	–	–	1.9 ± 1.5	–
Time above range (%)	–	–	33.7 ± 13.5	–
Glucose management indicator (%)	–	–	7.1 ± 0.5	–

Abbreviations: Ctrl, control group; eGFR (CKD‐EPI), estimated glomerular filtration rate calculated using the Chronic Kidney Disease Epidemiology Collaboration equation; HbA1c, glycated haemoglobin; HR, heart rate; MAP, mean arterial pressure; T1D, type 1 diabetes mellitus.

### 3.2 Analysis of fluid shift

#### 3.2.1 Thoracic

The mean thoracic blood shift, assessed by segmental bioimpedance, was not different between Ctrl and T1D participants (group effect, *P* = 0.731) nor between sexes (sex effect, *P* = 0.279). It was significantly higher during LBNP compared with HUT (*P* = 0.0171; η_p_^2^ = 0.277) and was significantly increased (*P* < 0.001; η_p_^2^ = 0.785) when both HUT angle and LBNP pressure were increased, with blood shifts going from 184.4 ± 82.4 to 430.1 ± 200.1 mL during HUT and from 194.8 ± 97.5 to 560.5 ± 160.8 mL during LBNP (Figure 4). *Post hoc* analysis of the interaction condition × intensity (*P* = 0.0397) highlighted the differences in blood shift between HUT and LBNP at the highest intensity (*P* = 0.0244, Cohen's *d* = 0.743, moderate effect). However, this analysis also outlined equivalent thoracic blood shift between: 22° of HUT and 10 mmHg during LBNP (*P* = 1.000; Cohen's *d* = 0.0475, trivial); 42° of HUT and 20 mmHg during LBNP (*P* = 1.000; Cohen's *d* = 0.129, trivial); and 80° of HUT and 35 mmHg during LBNP (*P* = 1.000; Cohens’ *d* = 0.0428, trivial).

#### 3.2.2 Abdomen

The mean abdominal blood shift was not different between Ctrl and T1D participants (*P* = 0.739) nor between sexes (*P* = 0.163). The mean abdominal blood shift was significantly different between HUT and LBNP (*P* < 0.001; η_p_^2^ = 0.930). The significant interaction (condition × intensity *P* < 0.001) and the *post hoc* analysis showed a progressive increase of blood in the abdomen during HUT, with an increase of TBV in the area from 292 ± 243 to 836 ± 568 mL (effect size *d* ranging from 0.483 to 1.222, HUT 22° vs. others) and a progressive decrease during LBNP with a decrease in blood volume in the area from 491 ± 351 to 951 ± 594 mL (effect size *d* ranging from 0.440 to 1.337, 10 mmHg vs. others) (Figure 4).

#### 3.2.3 Pelvis

The mean pelvic blood shift was not different between Ctrl and T1D participants (*P* = 0.812) nor between sexes (*P* = 0.560). The pelvic blood pooling was significantly increased by the intensity of the orthostatic stress (*P* < 0.001; η_p_^2^ = 0.572), but it was significantly different between HUT and LBNP (*P* < 0.001; η_p_^2^ = 0.661), with a higher blood pooling during LBNP compared with HUT. There was no interaction between condition and intensity (*P* = 0.0813). During HUT, the pelvic blood shift increased from 41.3 ± 37.4 to 82.46 ± 50.8 mL, and it increased from 93.3 ± 62.9 to 230.1 ± 136.1 mL during LBNP (Figure 4).

#### 3.2.4 Leg

The mean leg blood shift was not different between Ctrl and T1D participants (*P* = 0.662) nor between sexes (*P* = 0.733). The leg blood shift was significantly increased by the intensity of the orthostatic stress (*P* = 0.0266; η_p_^2^ = 0.349) in a similar way during HUT and LBNP (*P* = 0.175). The absence of interaction between factors (*P* = 0.846) reflects the same pattern in responses between HUT and LBNP when increasing the intensity (Figure 4).

### 3.3 Haemodynamic responses at equivalent preload reduction between HUT and LBNP

Given that haemodynamic parameters are highly dependent on the cardiac preload, the comparison between HUT and LBNP was based on the intensities inducing equivalent TBV shift previously determined. These equivalences are based on the smallest effect size of interest (Riesthuis, 2024), namely, baseline pre‐tilt vs. baseline pre‐LBNP: 22° of HUT vs. 10 mmHg of LBNP (Cohen's *d* = 0.0475); 42° of HUT vs. 20 mmHg of LBNP (Cohen's *d* = 0.129); and 80° of HUT vs. 35 mmHg of LBNP (Cohen's *d* = 0.0428).

An analysis of the consecutive baselines can be found in the Supplementary material (Supplementary results section and Figure S1).

#### 3.3.1 Arterial blood pressure

**FIGURE 5:**
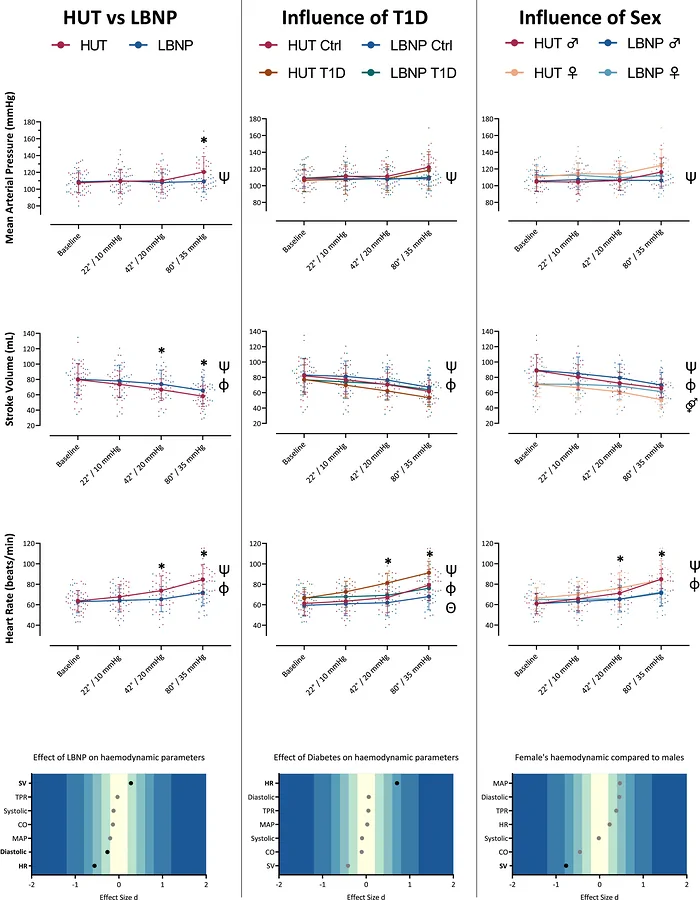
Graphical representation of haemodynamic parameters during HUT and LBNP at equivalent modification of thoracic blood shift. The first row represents MAP responses, the second row SV and the third row HR. The left column represents the overall analysis, the middle column the analysis differentiating the participants according to their group, and the right column represents the analysis differentiating the participants according to their sex. Error bars indicate the SD. ^φ^Significant effect of the LBNP. ^ψ^Significant effect of the orthostatic stress intensity. ^*^Significant difference between modalities at a specific intensity. ^θ^Significant difference between Ctrl and T1D participants. ^ψ^Significant difference between males and females. The bottom row represents effect sizes of haemodynamic parameters. Haemodynamic parameters are ordered from the largest positive effect size to the largest negative effect size. Grey dots represent a non‐significant difference. Black dots represent a significant effect of the main variable (LBNP, sex or diabetes). The colour gradient represents the interpretation of the effect size (0 < *d* < 0.2, trivial difference; 0.2 < *d* < 0.4, very low difference; 0.4 < *d* < 0.6, low difference; 0.6 < *d* < 0.8, moderate difference; 0.8 < *d* < 1.2, high difference; 1.2 < *d* < 2, very high difference). Abbreviations: CO, cardiac output; Ctrl, control; HR, heart rate; HUT, head‐up tilt; LBNP, lower‐body negative pressure; MAP, mean arterial blood pressure; SV, stroke volume; T1D, type 1 diabetes mellitus; TPR, total peripheral resistance.

The mean arterial pressure (MAP) showed no difference between HUT and LBNP (*P* = 0.0104), but seemed to be increased by the orthostatic stress intensity (*P* < 0.001; η_p_^2^ = 0.278). The *post hoc* analysis of the interaction of condition × intensity (*P* < 0.001) outlined that all conditions and intensities were comparable except for 80° of HUT, which induced higher blood pressure in comparison to all other experimental conditions (*P* < 0.001, Cohen's *d* > 0.709). There was no difference between Ctrl and T1D participants (*P* = 0.937) nor between sexes (*P* = 0.156) (Figure 5).

##### 3.3.1.1 Total peripheral resistance

The TPR was increased by the intensity of orthostatic stress (*P* < 0.001; η_p_^2^ = 0.234), with no difference between HUT and LBNP (*P* = 0.729) and no effect of diabetes (*P* = 0.882) or sex (*P* = 0.280). No interaction was highlighted by the statistical analysis (*P* = 0.328).

##### 3.3.1.2 Heart rate

At equivalent shifts in TBV, HR was increased by the orthostatic stress intensity (*P* < 0.001; η_p_^2^ = 0.765) and was higher during HUT compared with LBNP (*P* < 0.001; η_p_^2^ = 0.696). *Post hoc* analysis of the interaction of condition × intensity (*P* < 0.001) showed that during HUT, HR was not different compared with LBNP at rest and during the first intensity (*P* > 0.286), but was higher during HUT with the two highest intensities (42° vs. 20 mmHg and 80° vs. 35 mmHg, *P* < 0.001, Cohen's *d* > 0.725, moderate difference). The T1D participants exhibited higher HR compared with the Ctrl group (*P* = 0.0483), with no significant interactions with the other factors (condition, intensity). Sex showed no effect on HR (*P* = 0.504) (Figure 5).

##### 3.3.1.3 Stroke volume

At equivalent shifts in TBV, SV decreased with increasing orthostatic stress intensity (*P* < 0.001; η_p_^2^ = 0.777). Overall, SV values were higher during LBNP compared with HUT (*P* = 0.00225; η_p_^2^ = 0.317). The *post hoc* analysis of the condition × intensity interaction (*P* = 0.00980) showed that HUT reduced SV significantly between each intensity (*P* < 0.00482, Cohen's *d* > 0.368), whereas during LBNP only the highest intensity differed from the preceding level. Furthermore, the *post hoc* analysis showed that SV was different between HUT and LBNP during the two highest intensities (42° vs. 20 mmHg, *P* = 0.00660, Cohen's *d* = 0.422; 80° vs. 35 mmHg, *P* = 0.0163, Cohen's *d* = 0.392).

Specific analysis revealed no significant effect of T1D on SV (*P* = 0.240). However, females exhibited overall lower SV values than males (*P* = 0.0360, Cohen's *d* = 0.767), together with a significant sex × intensity interaction (*P* = 0.0354). In males, SV decreased significantly even at low orthostatic stress (baseline vs. 22°/10 mmHg, *P* = 0.0222; Cohen's *d* = 0.380), whereas in females the SV remained unchanged between baseline and the lowest intensity (*P* = 1.000) and between the first two intensity levels (22°/10 mmHg vs. 42°/20 mmHg; *P* = 0.573) (Figure 5).

##### 3.3.1.4 Cardiac output

At equivalent shifts in TBV, CO was significantly reduced with intensity (*P* = 0.0128; η_p_^2^ = 0.163) and showed a statistical interaction between condition and intensity (*P* = 0.0127). *Post hoc* analysis highlighted that CO was not modified with the angle of the HUT (with a maximum Cohen's *d* of 0.0722), whereas it was significantly reduced during LBNP (baseline vs. 35 mmHg, *P* < 0.001, Cohen's *d* = 0.353, very low difference). Consequently, CO was significantly higher during HUT at 80° compared with LBNP at 35 mmHg (*P* = 0.0131, Cohen's *d* = 0.389).

No difference was observed between Ctrl and T1D participants (*P* = 0.787). Specific analysis for sex highlighted no absolute difference because of sex (*P* = 0.233) but a three‐way condition × intensity × sex interaction (*P* = 0.00925) outlined that the difference in CO between 80° and 35 mmHg occurred only in male participants (*P* < 0.001, Cohen's *d* = 0.783 for males; *P* = 1 for females).

## 4 DISCUSSION

In the present study, we compared haemodynamic and fluid redistribution responses to graded HUT and LBNP, using changes in thoracic impedance as an index of central hypovolaemia. For equivalent reductions in thoracic impedance, SV decreased more during HUT than during LBNP, whereas HR was consistently elevated during HUT, thereby preserving CO. MAP and TPR remained stable across modalities. These differences are likely to reflect distinct regional pooling patterns, mainly splanchnic during HUT and pelvic during LBNP, highlighting the influence of body orientation and gravitational loading on cardiovascular adjustments (Taneja et al., 2007). Sex and T1D modulated these responses further: females displayed lower SV but preserved vascular regulation, whereas participants with T1D exhibited uniformly elevated HR, consistent with early alterations in autonomic–cardiac control that might prove clinically relevant. Collectively, our findings suggest that although HUT and LBNP result in comparable central hypovolaemia, they provoke mechanistically distinct strategies to maintain cardiovascular stability, differences that are shaped further by sex and early cardiometabolic disturbance.

### 4.1 Body fluid redistribution

Although measurements of thoracic impedance suggest that HUT and LBNP can induce comparable reductions in TBV, our findings emphasize clear differences in both thoracic and regional fluid redistribution. Consistent with Taneja et al. (2007) and supported by our findings, redistribution patterns were markedly different in the abdominal and pelvic regions: LBNP promoted splanchnic emptying and greater pelvic pooling, whereas HUT tended to increase abdominal blood content. Crucially, our protocol diverged from that of Taneja et al. (2007) by randomizing the order of intensities instead of applying sequentially increasing tilt or negative pressure, thereby limiting cumulative physiological drift. This approach revealed distinct patterns of TBV shift between the two modalities, particularly at higher intensities. With increasing LBNP, TBV declined linearly, whereas during HUT the reduction followed the sine of the tilt angle, leading to a flattening of the curve between 58° and 80°. At the same time, gravitational pooling during HUT occurred predominantly in the abdominal compartment, whereas negative pressure facilitated unloading of this region during LBNP. The combination of a greater thoracic blood shift and abdominal emptying during LBNP resulted in significant pelvic pooling, markedly higher than that observed during HUT.

Thus, the splanchnic region functions as the primary reservoir during HUT, whereas the pelvic region assumes this role during LBNP. This reflects the distinction between a natural gravitational force acting homogeneously on the body and an artificial and forced differential pressure on the lower half of the body. Finally, contrary to Taneja et al. (2007), we observed no significant differences in leg blood volume between modalities. This discrepancy might be explained by our use of a saddle instead of a footrest, which minimized lower‐limb muscle activity and ensured more comparable conditions between HUT and LBNP.

### 4.2 Differences in cardiovascular regulation between HUT and LBNP

Although both HUT and LBNP are commonly used to simulate orthostatic stress, the comparative studies are equivocal. Although these comparisons are highly limited, early work by Patwardhan et al. (1995) reported comparable autonomic activation during HUT and LBNP, with sympathetic nerve activity stimulated in both conditions (Patwardhan et al., 1995) following the typical responses to a reduced central blood volume and hypotension (Goswami et al., 2019). Conversely, other studies have identified notable differences in cardiovascular regulation between the two modalities. Specifically, HUT appears to increase vascular resistance primarily in the lower body, whereas LBNP induces a more uniform rise in vascular resistance, and HR responses are typically greater during HUT (Kitano et al., 2005). These findings concur with more recent work suggesting that the lower‐body vasculature disproportionately contributes to baroregulation, whereas upper‐body regions play a greater role in thermoregulation (Fisher et al., 2025). Beyond peripheral vascular responses, Bronzwaer et al. (2017) demonstrated that cerebral blood flow is lower during HUT compared with LBNP, which might explain, in part, the enhanced tachycardic response.

In the present study, for equivalent reductions in thoracic impedance, CO was maintained during HUT despite a more pronounced reduction in SV, indicating a proportionally greater chronotropic adjustment. This is likely to reflect both baroreflex compensation for the additional SV reduction and the contribution of gravitational and postural factors, such as vestibular‐driven sympathetic activation, which is absent during LBNP, augmenting HR to counteract orthostatic stress (Carter & Ray, 2008). The associated rise in diastolic pressure, despite similar TPR, further supports differential modulation of vascular tone and HR control between conditions.

Collectively, these differences highlight that, even when matched for impedance‐based preload reduction, HUT and LBNP elicit distinct cardiovascular adjustment strategies. This divergence might arise from differences in regional pooling that impact venous return (Taneja et al., 2007) or from an additional hydrostatic gradient along the cardiac axis during HUT (Pantalos et al., 2005). Such distinctions are crucial when LBNP is used as an analogue for HUT in contexts requiring supine positioning (e.g., MRI) (Kimmerly et al., 2005), although LBNP remains valuable for isolating baroreflex‐mediated responses without vestibular stimulation.

### 4.3 Methodological considerations for matching preload between HUT and LBNP

The present findings are consistent with previous reports suggesting that segmental bioelectrical impedance is a valuable non‐invasive surrogate for estimating central blood volume and preload status (Krantz et al., 2000; Matzen et al., 1991), thereby supporting the selected LBNP intensities (−10, −20 and −35 mmHg) as analogues for graded orthostatic stress (22°, 42° and 80°), originally recommended by König et al. (1993).

However, our results also indicate that ‘preload‐equivalent’ conditions based on thoracic impedance might conceal meaningful physiological differences. Despite equivalent changes in estimated central volume and mean arterial pressure, SV and CO differed between modalities, suggesting that impedance‐derived preload does not fully capture the complex interplay between venous compliance, regional pooling and cardiac filling pressures. Thoracic impedance reflects pulmonary, aortic and intracardiac blood volumes, all of which influence the signal but do not necessarily correspond to true cardiac preload. Although invasive right‐heart catheterization with direct central venous pressure measurement remains the reference standard, it is unsuitable for studies in healthy participants. In this context, non‐invasive imaging, particularly echocardiography, provides a practical alternative to quantify cardiac volumes and assess filling dynamics in varying gravitational and postural conditions. The better‐preserved SV during LBNP suggests that the intraventricular hydrostatic pressure gradient described by Pantalos et al. (1998, 2005) imposes an additional gravitational load during HUT that might constrain ventricular ejection. Future work from our group will integrate echocardiographic indices of preload and cardiac adaptation within the same experimental framework to refine the interpretation and validity of impedance‐based measurements.

### 4.4 Specific population analysis

#### 4.4.1 T1D

The inclusion of young participants with T1D provides a valuable human model to explore early cardiovascular dysregulation. Beyond emerging myocardial alterations (Hodzic et al., 2016), T1D is associated with a high prevalence of subclinical autonomic dysfunction, which can occur early in the disease course and frequently remains undetected (Hamdaoui‐Ayad et al., 2012; Sundkvist et al., 1980). In particular, early cardiac autonomic neuropathy is characterized by a reduction in parasympathetic tone, leading to decreased heart rate variability and relative sympathetic predominance, even before overt cardiovascular disease becomes apparent (Williams et al., 2022). In this context, studying young adults with uncomplicated T1D allows investigation of early alterations in autonomic cardiovascular control while minimizing confounding structural heart disease. Accordingly, participants with known cardiac dysfunction were excluded to isolate early functional and neuroregulatory mechanisms specifically, rather than established myocardial pathology.

To our knowledge, only one previous study has examined acute haemodynamic responses to LBNP‐induced hypovolaemic stress (up to −30 mmHg) in 15 young women with T1D, focusing mainly on vascular responses and showing impaired capillary fluid absorption and reduced vasoconstrictor responses in comparison to healthy matched control subjects (Lindenberger et al., 2013). In contrast, despite comparable age and exposure to higher orthostatic loads in our study (up to −35 mmHg and 80°), participants with well‐controlled T1D maintained stable haemodynamics, with SV, CO, MAP and TPR comparable to control subjects. The only consistent difference was an elevated HR, suggesting an early compensatory mechanism that preserves CO. This could reflect an early stage of autonomic imbalance, in which sympathetic drive compensates for incipient cardiac dysfunction, potentially reducing HR reserve and orthostatic tolerance under more extreme stress. This pattern supports the view that early diabetic autonomic dysfunction is characterized by early parasympathetic withdrawal and an exaggerated chronotropic drive, preceding overt sympathetic or vasomotor impairment (Pop‐Busui, 2012). Physiologically, this might represent a transitional phase, in which compensatory mechanisms maintain haemodynamic stability under moderate orthostatic stress. Clinically, our findings underscore the potential value of dynamic orthostatic testing to detect early reductions in autonomic reserve in otherwise well‐controlled T1D patients, before the emergence of overt orthostatic intolerance or blood pressure dysregulation.

Notably, two participants with T1D experienced clinical events during the protocol, including thoracic pain during LBNP (−20 mmHg) without evidence of hypotension or myocardial ischaemia, and a presyncopal episode during HUT (80°), respectively. Although these participants were not included in the final analysis owing to incomplete datasets resulting from early discontinuation of the protocol, these observations might suggest early individual variability in orthostatic tolerance within this population, despite preserved average haemodynamic responses. More broadly, our results should be interpreted in the context of the experimental models used. Although HUT represents a well‐established approach for investigating orthostatic stress, it remains an experimental model that does not fully replicate real‐life orthostatic conditions, particularly with respect to muscle activation and neurovascular responses.

Future longitudinal studies integrating haemodynamic, autonomic and echocardiographic markers will be essential to determine whether this compensatory pattern precedes measurable declines in orthostatic regulation or cardiac performance.

#### 4.4.2 Sex effect

The sex‐related differences observed here, namely higher SV in males, are consistent with established anatomical and functional dimorphisms, in that men typically exhibit larger cardiac dimensions and blood volumes (Lang et al., 2015). However, our findings also highlight that despite the absence of differences in HR, MAP and TPR responses, implying preserved compensatory capacity, a functional divergence in cardiovascular control emerges. Although CO responses to HUT and LBNP were comparable in females, males exhibited higher CO during HUT than during LBNP at equivalent reductions in TBV, suggesting sex‐specific regulatory strategies. Prior research indicates that women display lower sympathetic vasoconstrictor responsiveness and rely more heavily on vagal modulation, contributing to their lower orthostatic tolerance relative to men (Koenig & Thayer, 2016; Shoemaker et al., 2001). Our findings extend this knowledge by demonstrating that sex‐related differences in cardiovascular control persist even when preload reductions are standardized, implying that factors beyond sympathetic and anthropometric differences, such as body orientation and gravitational loading, might modulate cardiovascular regulation differently in males and females.

### 4.5 Limitations

This study has several limitations that should be acknowledged. First, thoracic impedance was used as a surrogate for central blood volume and preload. Although this technique enables continuous and non‐invasive monitoring, it provides only an indirect estimation of cardiac preload and might be affected by regional fluid shifts and changes in thoracic conductivity. The same limitation can be formulated for the blood pressure measurement. Although finger plethysmography is widely used and validated in the context of orthostatic stress (Lucci et al., 2023), it is also known that its reliability can drop in the event of high modification of vascular responses, especially concerning absolute values (Bos et al., 1995; Dyson et al., 2010; Raamat et al., 2001; Wesseling et al., 1993).

Second, although the use of a saddle minimized lower‐limb muscle activation and improved comparability between HUT and LBNP, it also induced some discomfort during the 80° tilt, which might have influenced sympathetic activation. Although the duration of each stage was sufficient to reach a steady state in cardiovascular responses, longer exposures might be required to achieve full equilibrium in regional fluid redistribution, particularly in the splanchnic and interstitial compartments. A longer duration of LBNP (e.g., 30–60 min) might allow additional slow processes, such as interstitial fluid shifts and progressive vascular adaptations, to develop. Therefore, our protocol primarily reflects the early steady state of venous capacitance and blood redistribution and might not fully capture the later plateau associated with prolonged orthostatic stress. This should be considered when comparing our findings with studies using extended durations of LBNP.

Third, although participants with T1D were well controlled and free of clinical neuropathy, our findings cannot be generalized to individuals with advanced autonomic dysfunction. The sample size was modest, and the study was not powered to detect subtle interaction effects between sex and diabetes. Finally, the absence of direct autonomic or cerebral blood flow measurements prevents detailed assessment of neural or cerebrovascular contributions to the observed cardiovascular responses.

## 5 CONCLUSION

By combining HUT and LBNP across graded levels of orthostatic stress, in this study we established dose–response relationships linking gravitational load to key cardiovascular indices, providing a reference framework for simulating circulatory responses across the continuum of 0–1*g*. Although both modalities induced comparable reductions in central blood volume, their physiological signatures diverged. HUT induced gravitational forces that promoted splanchnic pooling and a compensatory tachycardia capable of maintaining CO across tilt angles of ≤1*g*. In contrast, LBNP induced pelvic pooling through lower‐body suction while excluding gravitational loading, thereby isolating baroreflex‐mediated responses without modifying cardiac orientation. The finding that SV differs despite similar reductions in TBV underscores the complexity of orthostatic regulation, in which preload, autonomic adjustments and vascular compliance interact dynamically. Appreciation of these distinctions is crucial when interpreting orthostatic responses in individuals with altered cardiac or autonomic control. Overall, this dual modality approach refines our understanding of how gravity shapes cardiovascular regulation and offers a more robust methodological basis for future investigations in both clinical and spaceflight contexts.

## AUTHOR CONTRIBUTIONS

Victorien Faivre‐Rampant: Writing—original draft, Writing—review and editing, Investigation, Data curation, Formal analysis, Methodology, Project administration, Visualization. Perrine Larmet: Investigation, Writing—review and editing. Jules Warnier: Data curation, Formal analysis, Investigation, Writing—review and editing. Damien Garcia: Data curation, Resources, Validation, Writing—review and editing. Michael Joubert: Resources, Formal analysis, Writing—review and editing. Damian Miles Bailey: Validation, Writing—review and editing. Igor Mekjavic: Validation, Supervision, Writing—review and editing. Hervé Normand: Conceptualization, Data curation, Investigation, Methodology, Project administration, Resources, Supervision, Validation, Writing—review and editing. Amir Hodzic: Conceptualization, Data curation, Funding acquisition, Investigation, Methodology, Project administration, Resources, Supervision, Validation, Writing—original draft, Writing—review and editing. All authors approved the final version of the manuscript and agree to be accountable for all aspects of the work in ensuring that questions related to the accuracy or integrity of any part of the work are appropriately investigated and resolved. All persons designated as authors qualify for authorship, and all those who qualify for authorship are listed.

## CONFLICT OF INTEREST

D.M.B. is Editor‐in‐Chief of *Experimental Physiology* and outgoing Chair of the Life Sciences Working Group and outgoing member of the Human Spaceflight and Exploration Science Advisory Committee to ESA. D.M.B. is a current member of the Space Exploration Advisory Committees to the UK and Swedish National Space Agencies.

## GENERATIVE AI STATEMENT

For this study, artificial intelligence was used according to Wiley's policy. Generative artificial intelligence (GPT 5) was not used for any text generation; it was used only as a reader and reviewer, giving comments to the fill the gaps and improve the language and readability of the paper.

## Supporting information



Supplementary Information

## Data Availability

The datasets generated and analysed during the present study are available from the corresponding author on reasonable request.
