# Supplementary material for: Haemodynamic responses to head‐up tilt versus lower‐body negative pressure in type 1 diabetes
Source: Exp Physiol. 2026 Jul 28;111(9):4089–102. doi: 10.1113/EP093588 (PMC13409011; doi:10.1113/EP093588)
Supplement: Supplementary file 1 [file EPH-111-4089-s001.docx]

**SUPPLEMENTARY MATERIAL**

Haemodynamic responses to head-up tilt versus lower body negative pressure in Type 1 diabetes

**Supplementary Methods**

**Table S1**. Inclusion, non-inclusion, and exclusion criteria of the experiment.

| Inclusion criteria | For both groups:   - Participant affiliated with the French national health insurance system - Signed informed consent - Age ≥ 18 years old   For T1D participants:   - Diagnosed with Type 1 diabetes for at least 10 years - Followed at the Caen University Hospital Center (CHU de Caen) - Equipped with a Continuous Glucose Monitoring system (CGM) |
| --- | --- |
| Non-inclusion criteria | - Physically trained individuals (≥ 6 hours per week of sustained exercise for more than 6 months) - Presence of chronic cardiovascular disease or ongoing cardiovascular treatment - Personal history of chemotherapy and/or thoracic radiotherapy - Presence of neurocerebral and/or spinal disorders - Known orthostatic intolerance - Type 1 diabetes without CGM system - Type 1 diabetes with known end-organ damage - Participant in another biomedical research protocol (during the present study or within the 3 months preceding inclusion). - Pregnant, breastfeeding or postpartum women - Adults under legal protection (guardianship, curatorship), unable to provide informed consent, or deprived of liberty. |
| Exclusion criteria | - Echocardiographic and/or 12-lead electrocardiogram abnormalities detected during baseline screening suggesting underlying heart disease. - Inadequate image quality for quantitative echocardiographic analysis. |

**Supplementary Results**

### **Evolution of baseline hemodynamic parameters throughout the protocol**

To assess potential changes in baseline cardiovascular parameters over the course of the protocol, hemodynamic measurements obtained at each baseline preceding the graded HUT and LBNP exposures were compared. Mean arterial blood (MAP) pressure increased throughout the experiment (p<0.001). Post hoc analyses revealed a marked rise after the first condition, followed by minimal or no further increases in subsequent stages. No significant effects of diabetes (p = 0.936) or sex (p = 0.312) were observed on MAP responses. The total peripheral resistance (TPR) was gradually increased along the experiment (p<0.001) with no effect of the diabetes (p=0.889) or sex (p=0.314).

The heart rate (HR) was reduced throughout the experiment (p<0.001), and the post hoc analysis showed that only the first baseline was different from the others, likely reflecting the attenuation of anticipatory stress after the initial exposure. The baseline HR was not affected by diabetes (p=0.059) or sex (p=0.773).

The stroke volume (SV) was stable throughout the experiment (p=0.754) with no effect of diabetes (p=0.354) or sex (p=0.210). The cardiac output followed HR pattern and was reduced throughout the experiment (p=0.001), the post-hoc analysis showed that only the first baseline was different from the others. Diabetes and sex did not induce any effect on baseline HR (p=0.926 and p=0.087, respectively).

**Figure S1**. Graphical representation of haemodynamic parameters during the successive baseline periods throughout the experiment protocol. Data are presented as mean ± SD for all participants (n = 30). # Expresses a significant effect of the consecutive conditions on the baseline.
